# The specific needs of patients following sepsis: a nested qualitative interview study

**DOI:** 10.3399/bjgpopen17X100725

**Published:** 2017-01-09

**Authors:** Sabine Gehrke-Beck, Mareike Bänfer, Nina Schilling, Konrad Schmidt, Jochen Gensichen, Christoph Heintze

**Affiliations:** 1 GP, Charité Universitätsmedizin Berlin, Institute of General Practice, Berlin, Germany; 2 Physician, Charité Universitätsmedizin Berlin, Institute of General Practice, Berlin, Germany; 3 Medical Student, Charité Universitätsmedizin Berlin, Institute of General Practice, Berlin, Germany; 4 GP, Jena University Hospital, Friedrich-Schiller-University, School of Medicine, Institute of General Practice and Family Medicine, Jena, Germany; 5 GP and Professor, Institute of General Practice, Medical Center of the University of Munich, Munich, Germany; 6 GP and Professor, Charité Universitätsmedizin Berlin, Institute of General Practice, Berlin, Germany

**Keywords:** primary health care, aftercare, patient education as topic, sepsis, professional delegation, qualitative research

## Abstract

**Background:**

Survivors of sepsis suffer from multiple critical disease sequelae when discharged to primary care. There is a lack of structured aftercare programmes and case managers may be helpful in caring for patients with chronic critical disease.

**Aim:**

To gain insight into the functioning of a structured aftercare programme for post-sepsis patients in general practice.

**Design & setting:**

A qualitative study using semi-structured interviews with patients and GPs across Germany who participated in an randomised controlled trial of a structured aftercare programme for post-sepsis patients, which included patient education and case manager monitoring.

**Method:**

Qualitative interviews with 19 patients and 13 GPs were audiorecorded, transcribed verbatim, and analysed using qualitative content analysis.

**Results:**

Patients appreciated the information given in the patient education session, but some disliked it because it reminded them of their serious illness. GPs appreciated patient education because well-informed patients are more likely to participate in follow-up. Patients appreciated the case monitoring because it made them feel safer and more cared for and helped them reflect on their health issues. However, some patients felt uncomfortable with the regular questioning. GPs appreciated the case management programme because they received regular clinical information. However some GPs were wary of the clinical relevance of the information, the delegation of the patient to the nurse, and efficiency of time. Both patients and GPs requested more clinical support, such as easier access to psychotherapists.

**Conclusion:**

In general, both patients and their GPs appreciated patient education and monitoring following sepsis. Patients’ retrospections and worries about their serious illness need to be considered.

## How this fits in

After discharge from hospital with sepsis, patients experience long-term physical and psychological sequelae of their illness. No aftercare schemes are established so far and follow-up interventions in randomised controlled trials (RCTs) showed heterogeneous results. This interview study nested in an aftercare RCT, elicited the effectiveness of a case manager follow-up. Possible barriers that emerged were concerns about education for patients and delegation of tasks and care for GPs.

## Introduction

In an intensive care unit, sepsis is a common diagnosis with 4% of all ICU stays accounting for sepsis and 11% for severe sepsis.^1^ In primary care, the follow-up of a post-sepsis patient is not an everyday routine event and a GP can expect to see a patient for follow-up after sepsis two to three times per year.^1^

Post-sepsis patients can experience multiple sequelae. Nearly all patients discharged after critical care have impaired activity of daily life;^2^ up to 50% suffer from critical illness-like neuropathy and myopathy.^2^ Post-traumatic stress disorder (PTSD) and depression affect one-third of patients^2–5^ and long-term mortality after discharge is significantly increased.^6^ GPs need to initiate, monitor, and coordinate care for the complex needs of these patients after discharge. A structured aftercare programme could be helpful and support GPs but currently one does not exist. General hospital-based aftercare programmes have been evaluated with heterogeneous effects.^7^

The SMOOTH (Sepsis Survivors Monitoring and Coordination in Outpatient Healthcare) study evaluated a structured aftercare programme in primary care. The study included adult survivors of severe sepsis or septic shock according to International Classification of Diseases-10 (ICD-10) codes in 19 intensive care units in Germany. The structured aftercare included patient and GP education and patient monitoring by a case manager. Intensive care nurses were trained as case managers. The educational session for patients took place at their homes. The case manager provided information on sepsis sequelae and treatment options, and encouraged adherence to medication, physiotherapy, and a recommended diet. Monitoring consisted of one initial home visit and consecutive calls every 4 weeks for half a year. The results of the monitoring were sent to the liaison GP, who provided clinical decision support to the patient’s GP. Details of the RCT are published elsewhere.^8^ The study did not find improvement in mental health-related quality of life.^8^

The aim of this interview study was to elicit the views of patients and their GPs on the functioning of the case manager intervention.

## Method

### Design and participants

This was a qualitative, observational study using semi-structured interviews with patients and GPs from the SMOOTH study in Germany. Patients and GPs that had experienced the intervention for at least 8 months between April 2013 and March 2014 were interviewed. Maximum variation sampling using the criteria age, sex, and pre-existing chronic disease was conducted and saturation was reached after 19 patient interviews and 13 interviews with GPs.

### Data collection and analysis

Interview guides for patients and doctors were developed from a theoretical framework derived from literature research covering sepsis sequelae, sepsis aftercare, and case-manager intervention in other chronic conditions.^7–12^ This study reports the perspectives of patients and GPs on the case manager functioning in the intervention; that is, on the delivery of patient education and on the monitoring.

After written consent, two trained researchers from medical and nursing backgrounds conducted the interviews. Most patients chose to be interviewed at home (with one in a dialysis practice, and one in the workplace) and all doctors chose to be interviewed in their practice.

Interviews were audiorecorded, transcribed verbatim, and checked against the audiorecording by an independent researcher. Interviews were analysed using qualitative content analysis using Mayring.^13^ Patients and GPs interviews were analysed separately but preliminary results were discussed among the coders and new questions to the material evolved. The researchers started coding with a deductive codes framework derived from literature research. By analysing the text step-by-step, they inductively added and changed categories deriving from the material. The final coding framework (Box 1) was applied to all interviews. Intercoder reliability was assessed in two interviews in each group. Quotations were translated to from German to English for this article.

**Box 1.** Coding framework

**Patient perspective**			
**Patient education**	**Information**	Written information as reference for arising questions	
		Lack of interest	
		Refusal of information	Recall of burdensome memories
	**Education session**	No recall of education	
		Effect on health behaviour	
**Monitoring**	**Questioning**	Safety	
		Personal attention	
		Encourages personal reflection on health	
		Inconvenient	
		Recall of burdensome memories	
	**Care**	Helpful talking	
		Practical help	
		Lack of practical support and direct help	
	**Feedback of results**	Feedback not noticed	
		Feedback noticed, not discussed with GP	
		Results discussed with GP	
		GP considered monitoring results irrelevant	

**GPs’ perspective**			
**Patient education**	Not noticed		
	Considered unnecessary		
	Patient autonomy improves GP care		
**Monitoring**	**Effects on care**	Enhances patient wellbeing	
		Additional medical information	
		Unnecessary for uncomplicated patient	
		Not time saving	
	**Delegation**	Personal patient contact necessary	
		Time and money expenses	
		Burden for patients	
		Benefits of delegation	Additional perspective
			Additional home visit
			Active questioning
	**Implementation of therapy**	No problems	Problems expectes in similar patients in different circumstances (location, insurance status)
		Financial strains	
		Problems of avaibility	
		Problems of transport	

## Results

### Study population

Nineteen patients and 13 GPs were interviewed (see Table 1 for participant characteristics). Patient interviews lasted 18–67 minutes (mean 39 minutes), interviews with GPs lasted 12–28 minutes (mean 20 minutes).

**Table 1.** Participants characteristics

	Patients	GPs
Total	19	13
Male	12	
Female	7	
Mean age (range), years	61 (42–84)	54 (41–64)
Mean time working in practice (range), years	– (–)	19 (9–33)

### Patient education: the patient perspective

Some of the patients interviewed did not remember the education session or any information given to them at all, even when they were reminded of the circumstances. Of those who could recall the education session or the manual being handed over, some stated a lack of interest, as they felt quite well again:

‘That wasn’t necessary. Don’t know what they wanted with it. Don’t have a clue. Well, as I didn’t feel bad.’ (P11)

Some even refused the information that was given to them. They felt burdened as the issue of their illness was picked up again and made them remember their time at the intensive care unit:

‘Yes, I got a manual … it was somehow just too much for me then.’ (P14)

Some patients appreciated the information given in the education session and used the written manual provided later on as a source of information when needed:

‘The manual is very important to me. It’s in the living room. In case I want to look something up. And if I am unsure — I often get it out.’ (P10)

Some reported changing their health behaviour after they got more information and as a consequence, got more involved in the treatment of their illnes:

‘… with regard to that I may say that some things got to me, so I started again to rebuild me, especially regarding food, when it wasn’t tasty what should I say? You just eat five meals. Yes, you just eat five meals then. Ok.’ (P16)

### Patient education: the GP perspective

Some of the GPs interviewed did not know about or notice the patient education:

‘Well, I don’t know if he got some education … did I get any reports?’ (GP2)

Some considered patient education in this context as unnecessary, as patients were feeling well again or were well informed beforehand. A few GPs noticed theirs patient were well informed because of the education given to them by the case manager and acknowledged that it made the treatment easier:

‘I found that unusual, that he was so well-informed. I appreciated that. Well, that really helps.’ (GP9)

### Case manager monitoring: the patient perspective

Patients’ views of case manager monitoring concerned the monitoring visits and phone calls and the consequences they experienced in their care.

#### Experiences of questioning

Some patients reported that the repeated questioning by the case manager made them feel safe:

‘That was reassuring to me. That was reassuring — I felt in good hands.’ (P2)

And that they received personal attention:

‘Somehow, that was pleasant, as I was still at home, I still was on sick leave, when she phoned and asked whether I had half an hour. That was, well, somehow nice. I would say.’ (P13)

Some patients explained that the repeated questions made them think more about their health issues:

‘I could reflect on my own then, how I really felt. For that, it wasn’t bad actually … well, I considered it helpful. For me as a patient.’ (P18)

A few stated irritation with the monitoring process as it was inconvenient to them:

‘There was a short time, it simply was annoying to me. Yes. Let’s be honest. It was annoying to me.’ (P1)

Some found the monitoring wearing as they did not want to talk about their illness and did not want to be reminded of their time spent in the intensive care unit:

‘As I wanted to get done with it — well, that was always refreshed again, I had that feeling. As I said, I was sometimes so irritated, not with the woman, but as I didn’t want to talk about it anymore.’ (P13)

#### Experiences of care

Some patients felt cared for with the case manager monitoring as talking about their health issues and the counselling taking place was helpful:

‘It was pleasant, that one could talk to someone else, too. As sometimes I, as before Christmas, when sometimes I start again a depressive phase … that there is another someone. That helped me.’ (P15)

Some got practical support from the case manager in sorting out organisational matters:

‘When I approached her … when I had problems … with [*prolonging my*] sick leave. Yes, and then she at once was willing [*to help me*] to get a letter and a report written from.’ (P2)

A number of patients felt that the questioning only occurred to get study data and did not see the benefit of monitoring for them. They felt a lack of care and missed practical support:

‘The woman, from the study, you could tell her: look, I have a problem since then, yes. And she passed it on and I told her: my hip is aching. She passed that on. But there was nothing that was done.’ (P14)

#### Feedback to GPs

Many patients did not notice that the results of the case manager monitoring went back to their GP so that they could adapt treatment:

‘The feedback and the advice you were asking about I have noticed nothing, nothing negative, nothing positive. I would have liked to notice it, one way or another.’ (P7)

Some knew, that their GP got information from the study but that was not discussed with them:

‘Yes, he has these SMOOTH study papers lying around. I went last week to see him. Then I saw that paper lying around.’ (P3)

A few patients said that results were discussed with them:

‘She always said when I was there: yes, it arrived and actually everything was ok. There were no aspects that suggested that something should be done.’ (P18)

Some reported that the GP felt the monitoring was unnecessary:

‘Yes, he told me the lady [*case manager*] was there. And then he died laughing at the many questions.’ (P10)

### Case manager monitoring: the GP perspective

GPs reported that three main aspects of case manager monitoring evolved: effects, delegation, and problems of implementation of advised therapy.

#### Effects of regular case manager monitoring

A number of GPs assumed their patients felt cared for and safe when being monitored by a case manager and that that may help them getting better:

‘I do think so, I do think so, because it gave her some safety and kept her grounded, that simply there is somebody who pays attention and she knew, she comes again and that was enhancing her quality of life I think.’ (GP5)

Few GPs reported gaining additional information from the monitoring that was helpful for guiding or initiating therapy and that may have been missed otherwise:

‘At one point, it happened, I believe, the patient had more severe pain and that came as feedback from the study, the red flag came and what should I do … I must say, that I am not very keen on home visits and the patient, as I said, wasn’t mobile really. There was the contact with his wife and I went there when it got worse. But she didn’t report about that and he did not phone up here either and say, you have to come.’ (GP11)

Some GPs considered the monitoring unnecessary, as their patient had an uncomplicated course of disease:

‘No, nothing at all. Everything was calm and uncomplicated. Basically, after the rehabilitation therapy everything was uncomplicated and after that it remained uncomplicated … yes, well, he was really like he was before.’ (GP10)

Some considered the monitoring by a case manager as generally ineffective, as they felt that this task could not be delegated and they could rely only on what they see themselves:

‘… well, I think it doesn’t release me from my duties, well, for the patient it is certainly pleasant … I’d say for me as a GP … what the colleague did there, the talking, is detailed and specific for that condition, but well, I think, basically one doesn’t absolutely need it.’ (GP5)

#### Concepts of delegation

Some GPs expressed that any history taking and examination could only be done by doctors as the personal contact with the patient is most important:

‘I take all histories myself, the patients then can relate to me … and I know how the patient is. One also gets to know the character during the history taking and now when I only get a paper from somebody else, which is very thorough … I am not interested in that.’ (GP6)

Some noted the financial and time costs of the case manager monitoring:

‘We just don’t have the time to read through such folders for each patient.’(GP6)

One GP mentioned a possible burden for patients when an additional person is involved in their care:

‘Mostly, that [*involvement of another person*] is an excessive demand for elderly people and they feel it is a burden and don’t want that.’ (GP12)

Some GPs saw the benefits of involving a case manager in the follow-up of patients. Advantages they mentioned were a different point of view, additional home visits, and active questioning:

‘Yes, well, its probably not wrong to … that patients are monitored long-term and also questioned even independently or just independently from the doctor, because the doctor has a certain point of view, especially when he has known the patient a long time, well … this external business … can bring a new aspect to the matter. Well for the patient, it is, I believe, not wrong, such long-term-monitoring.’ (GP11)

#### Implementation of therapy

A number of GPs reported no problems in implementing the therapy necessary for patients following sepsis. Some added that this was the case if the patient was uncomplicated, privately insured, or because the practice was situated in a big city, but they assumed that there may have been problems otherwise:

‘Here in Berlin we are lucky, we have psychological care available and rehabilitation care, we have everything readily available here in Berlin. We are better off than a rural GP in [*small town*], where I come from.’ (GP10)

Some GPs reported financial limitations. German GPs have a limited budget for medication and physiotherapy and some found it difficult to care for those patients within these boundaries:

‘When it comes to physiotherapy, speech therapy, occupational therapy even when she has a severe critical illness, we are left alone, well that is for me the biggest problem … that we really want to treat the severely ill patients well and according to guidelines and that we don’t have the possibility to do it.’ (GP1)

Some mentioned problems finding a psychotherapist or rehabilitation clinic for their patient:

‘Well, he still has a post traumatic distress syndrome and he still is looking for a psychologist. I believe, he has now found one, but it isn’t definite, it is only a trial appointment. Here … it is very difficult to find something. (GP4)

Some experienced problems with transport for patients who were not able to walk:

‘As I said, [*the patient*] needs a rollator and a daily walk to physiotherapy is not reasonable for him, this they don’t understand, well, normally he should, there is an outpatient rehabilitation programme in … street, he would need to get transport there daily forth and back …’ (GP2)

## Discussion

### Summary

This qualitative study gained insights from patients and GPs into the case manager function in a programme for post-sepsis aftercare. Some patients reported a benefit from the patient education. Some changed their health behaviour and GPs noticed well-informed and motivated patients. Other patients had no recall of the education session. Reasons for that were not clarified completely, but problems brought up by patients were subjective and therefore some showed a lack of interest and a refusal of more information as it would bring back memories of their illness. Psychological burdens (such as PTSD or depression) may have hindered some patients from benefitting from patient education.

Patients and GPs appreciated the feeling of safety and personal attention provided by the monitoring of a case manager. As with patient education, some patients felt burdened with the repeated questioning as it recalled the difficult times of their illness. Some patients expressed the wish for more practical support with organisational issues Also, some GPs experienced organisational difficulties with the implementation of therapy.

GPs’ views on the delegation of monitoring tasks to a case manager were heterogeneous. Feedback of the monitoring results back to the GP and benefit for their treatment was not clear for a number of patients. While some GPs acknowledged the potential benefits and few received additional information about their patient in the study, some felt that the personal contact with patients could not be delegated and therefore considered the case manager monitoring unnecessary.

### Strengths and limitations

This study combined data from patients and GPs and therefore explored the phenomena from two perspectives. Socially desired answers may introduce bias, however it was observed throughout the interviews that patients and GPs felt free to criticise the interventions.

In the RCT, GPs were recruited when their patient was included in the study and few GPs refused. Some GPs declined an interview due to lack of time or interest, which may have caused selection bias of motivated GPs. Most patients did not give reasons for not taking part in an interview; they may have declined because they were still psychologically distressed, or because of time constraints and financial restrictions, so therefore a selection bias of healthier patients is also possible.

### Comparison with existing literature

An appreciation of case manager follow-up by patients and GPs has been found in other studies investigating case manager programmes in other chronic diseases and frail older people.^9–16^ Feelings of safety and control are expressed by patients in those programmes. Practical support by the case manager giving advice or solving practical problems, is experienced by patients in other studies^14^ and some of the patients in the SMOOTH trial. However, some patients in the current interview study wished for more practical support and felt left burdened with organisational issues; for example, organising specialist care. Similar experiences are expressed by patients in another sepsis aftercare study: patients in that trial considered that monitoring was a mere data collection with no benefit for their therapy.^17^ Problems with access to specialist care and follow-up treatment are also described in other studies.^15,18^ It is possible that a lack of therapeutic consequences resulting from the case manager monitoring contributed to the SMOOTH RCT^8^ showing no improvement for patients. This may be due to either ineffective communication of results back to the GPs or difficulties in initiating appropriate therapy, such as problems with access to specialist care.

Some of the interviewed patients felt uncomfortable with the patient education and the monitoring questioning as it reminded them of their time at the intensive care unit. They preferred not to stir up their memories of the severe illness. PTSD is common in sepsis survivors^19^ and therefore, psychological burdens might be prevalent in a fair number of sepsis survivors. In another intervention study, the nurses conducting a monitoring intervention reported that they felt like they were intruding, when asking their questions.^17^ Still, patient education should be available for all patients, as lack of knowledge and understanding of sepsis and the sequelae was shown to be common in patients and their care givers and is considered to be a barrier to adequate help seeking.^18^ Patients who did not feel ready to receive information about their illness and thereby enhance autonomy and involvement in their treatment may have impaired effectiveness of patient education in the SMOOTH trial.

### Implications for research and practice

The findings of this study provide information for GPs caring for post-sepsis patients and may support the planning of new interventions for sepsis aftercare.

This study shows that aftercare for patients following sepsis needs special consideration as psychological and physical sequelae are both common and need to be addressed. PTSD and depression are often not apparent and readily reported by the patient. In this study psychological distress was a barrier for some patients to get necessary information about their illness and to accept regular monitoring. Therefore, it is essential for GPs to understand and recognise psychological and psychiatric critical illness sequelae and address them. The availability of psychotherapy could also be a problem, as reported by some GPs in this study.

Researchers that plan aftercare schemes for post-sepsis patients should develop interventions to deal with existing psychological burdens such as PTSD. Tackling that condition could improve quality of life and enable patients to become more actively involved in their treatment and follow-up.

The effects of the monitoring by a case manager were assessed heterogeneously by the GPs. The patients’ experiences of the monitoring visits were mainly positive, but did they not notice effects on the GP care. Future interventions could involve making the results and the feedback more transparent. The monitoring results and feedback from the case manager could be sent additionally to the patient to increase transparency.

Some GPs in this study were reluctant to delegate, however in Germany delegation of patient contacts to a nurse or nurse practitioner is unusual and rarely done. A few GPs saw this as an advantage as they got additional and helpful patient information from the case manager; those GPs valued the monitoring highly. The quantitative data of the trial suggest that GP therapy was rarely altered by monitoring results. In the UK and US, as delegation of follow-up to qualified nurses or nurse practitioners and their involvement in patient care in chronic illness is more common and accepted, case manager monitoring would probably be better accepted and valued by GPs. Communication of results and treatment consequences would possibly be less affected by concerns and reservations of GPs. A better applicability of the intervention in UK and US is therefore likely and could lead to effectiveness by better acknowledging of the monitoring results and more frequent therapy adaptions.
